# Low Ambient Temperature Exposition Impairs the Accuracy of a Non-invasive Heat-Flux Thermometer

**DOI:** 10.3389/fphys.2022.830059

**Published:** 2022-03-04

**Authors:** Michela Masè, Andreas Werner, Gabriel Putzer, Giovanni Avancini, Marika Falla, Hermann Brugger, Alessandro Micarelli, Giacomo Strapazzon

**Affiliations:** ^1^Institute of Mountain Emergency Medicine, Eurac Research, Bolzano, Italy; ^2^Institute of Physiology, Center for Space Medicine and Extreme Environments Berlin, Charité – Universitätsmedizin Berlin, Corporate Member of Freie Universität Berlin, Humboldt-Universität zu Berlin, Berlin, Germany; ^3^German Air Force – Centre of Aerospace Medicine, Aviation Physiology Training Centre, Aviation Physiology Diagnostic and Research, Königsbrück, Germany; ^4^Department of Anaesthesiology and Intensive Care Medicine, Medical University of Innsbruck, Innsbruck, Austria; ^5^Department of Anaesthesia and Intensive Care, Santa Chiara Hospital, Trento, Italy; ^6^Centre for Mind/Brain Sciences, CIMeC, University of Trento, Rovereto, Italy; ^7^ITER Center for Balance and Rehabilitation Research (ICBRR), Rome, Italy

**Keywords:** body temperature, physiological monitoring, non-invasive devices, wearables, cold environments, hypothermia

## Abstract

**Background:**

Indirect core body temperature (CBT) monitoring from skin sensors is gaining attention for in-field applications thanks to non-invasivity, portability, and easy probe positioning. Among skin sensors, heat-flux devices, such as the so-called Double Sensor (DS), have demonstrated reliability under various experimental and clinical conditions. Still, their accuracy at low ambient temperatures is unknown. In this randomized cross-over trial, we tested the effects of cold temperature exposition on DS performance in tracking CBT.

**Methods:**

Twenty-one participants were exposed to a warm (23.2 ± 0.4°C) and cold (−18.7 ± 1.0°C) room condition for 10 min, following a randomized cross-over design. The accuracy of the DS to estimate CBT in both settings was assessed by quantitative comparison with esophageal (reference) and tympanic (comparator) thermometers, using Bland–Altman and correlation analyses (Pearson’s correlation coefficient, *r*, and Lin’s concordance correlation coefficient, *CCC*).

**Results:**

In the warm room setting, the DS showed a moderate agreement with the esophageal sensor [bias = 0.09 (−1.51; 1.69) °C, *r* = 0.40 (*p* = 0.069), CCC = 0.22 (−0.006; 0.43)] and tympanic sensor [bias = 2.74 (1.13; 4.35) °C, *r* = 0.54 (*p* < 0.05), CCC = 0.09 (0.008; 0.16)]. DS accuracy significantly deteriorated in the cold room setting, where DS temperature overestimated esophageal temperature [bias = 2.16 (−0.89; 5.22) °C, *r* = 0.02 (0.94), CCC = 0.002 (−0.05; 0.06)]. Previous exposition to the cold influenced temperature values measured by the DS in the warm room setting, where significant differences (*p* < 0.00001) in DS temperature were observed between randomization groups.

**Conclusion:**

DS accuracy is influenced by environmental conditions and previous exposure to cold settings. These results suggest the present inadequacy of the DS device for in-field applications in low-temperature environments and advocate further technological advancements and proper sensor insulation to improve performance in these conditions.

## Introduction

Core body temperature (CBT) is commonly defined as the temperature of the brain and major internal organs (Aschoff and Wever, 1958; Kobayashi et al., 1975; Fulbrook, 1997). CBT can be invasively measured at different sites, such as the pulmonary artery, esophagus, nasopharynx, bladder, and rectum (Strapazzon et al., 2014; Masè et al., 2021). The relative advantages and disadvantages of each measurement site have been reviewed in depth elsewhere (Fulbrook, 1997; Moran and Mendal, 2002; Lim et al., 2008; Taylor et al., 2014). As a significant limitation, these sites display some degree of invasivity, confining their use to specific clinical situations. To address these problem, other places, such as the ear canal and the skin surface, have been proposed, which may allow non-invasive, indirect estimation of CBT (Brinnel and Cabanac, 1989; Gallimore, 2004; Gunga et al., 2008; Kimberger et al., 2013; Strapazzon et al., 2014; Asadian et al., 2016; Masè et al., 2020). Methods for indirect CBT determination from the skin surface have gained attention thanks to non-invasivity, easy probe positioning, and small size. However, the recording of skin temperature implies a measurement at the interface of two different environments, that is, the human body and the external environment. Skin temperature may be affected by various environmental conditions, such as changes in ambient temperature, wind chill (Lankford and Fox, 2021), solar or heat radiation, humidity, and thermoregulatory responses, such as sweating and changes in local skin blood flow.

A promising approach for the indirect determination of CBT from the skin is represented by non-invasive heat-flux sensors, such as the so-called Double Sensor (DS) device (Gunga et al., 2008). The DS calculates CBT from two temperature sensors separated by an insulating disk with a known heat transfer coefficient (see “Study Design, Protocol, and Temperature Monitoring”). With respect to previous approaches (Fox and Solman, 1971), it needs neither a heating element nor a zero-heat-flux balance (Gunga et al., 2008). To date, various studies have suggested the reliability and validity of heat-flux approaches and DS technology under different environmental conditions, as well as in clinical conditions (Muravchick, 1983; Sakuragi et al., 1993; Gunga et al., 2008, 2009; Kimberger et al., 2009, 2013; Zeiner et al., 2010; Teunissen et al., 2011; Opatz et al., 2013; Xu et al., 2013; Guschlbauer et al., 2016; Mazgaoker et al., 2017; Mendt et al., 2017, 2021; Stahn et al., 2017; Gómez-Romero et al., 2019; Janke et al., 2021). However, despite the fact that the DS has been previously tested under challenging extreme situations, such as space flights and high ambient temperature (40°C; Gunga et al., 2008, 2009; Stahn et al., 2017), evidence is lacking on the reliability of DS temperature monitoring at low ambient temperatures (<0°C) and in the presence of rapid changes in ambient temperature.

Based on these premises, the present study aims to determine the capability of DS technology to assess CBT during exposure to low ambient temperatures. Performance was evaluated and compared with an esophageal reference sensor and a tympanic thermistor-based thermometer. We hypothesized that low ambient temperature could impact DS accuracy and determine carry-over effects on subsequent measurements.

## Materials and Methods

### Study Group

Participants were recruited from the local mountain rescue organization. They were instructed to fast for at least 6 h before testing. Clinical history and medical examination were conducted to exclude any acute or chronic conditions or abnormalities of the ear canal or upper airways. After an ear examination to assess external and middle ear integrity, cerumen was removed from the ear canal, if necessary.

### Study Design, Protocol, and Temperature Monitoring

**Figure 1:**
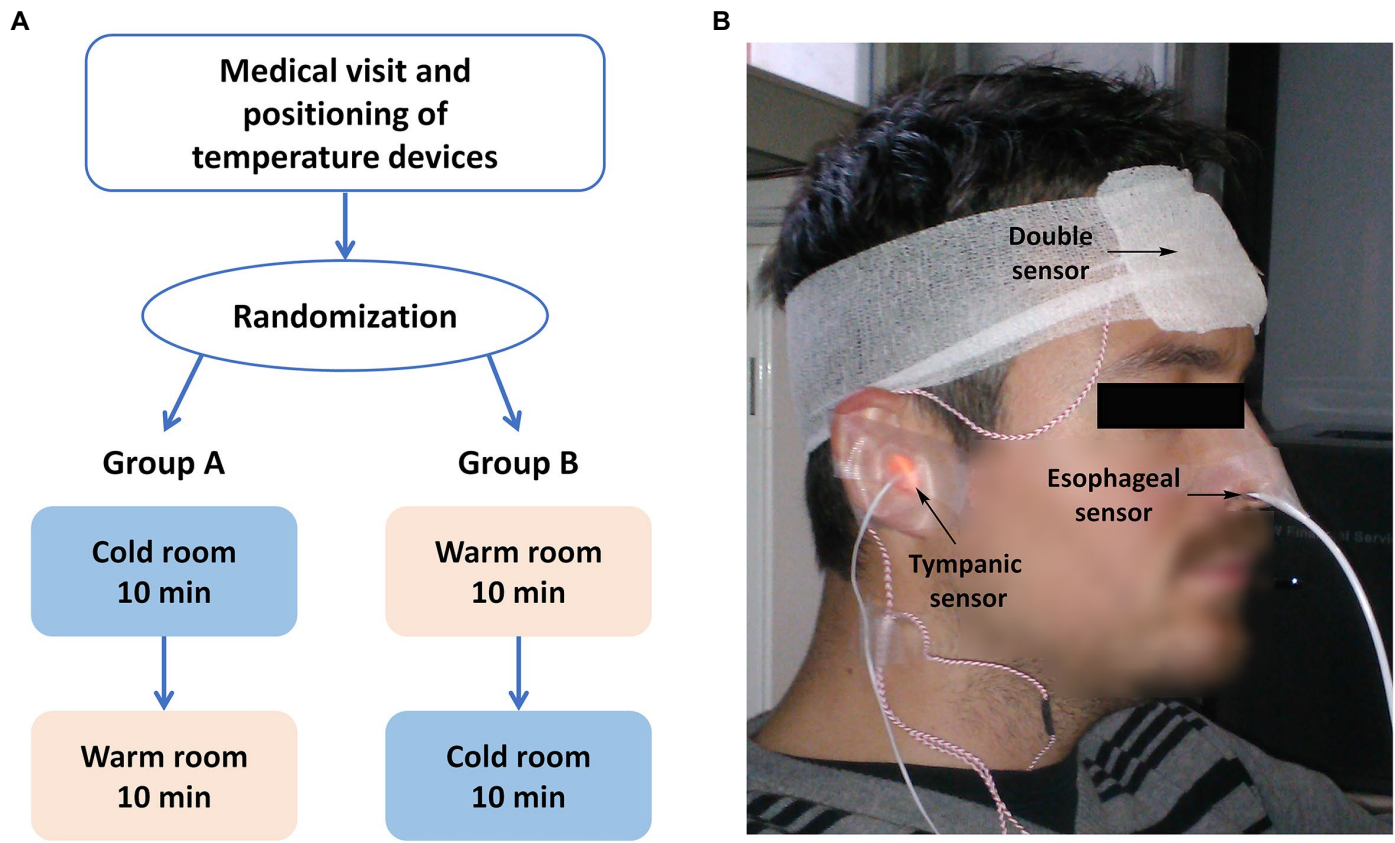
Study design **(A)** and experimental setup **(B)**. **(A)** The study was a randomized, cross-over study, where each participant underwent two sets of measurements, that is, measurements in a warm room (room temperature of 23.4 ± 0.4°C) and in a cold room (room temperature of –18.7 ± 1.0°C), and the order of the two conditions was the randomization variable. **(B)** Patients equipped with the devices for temperature monitoring: double sensor, esophageal, and tympanic thermometers.

The study was approved by the Ethics Committee review board of Bolzano, Italy (protocol n.70/2012) and conducted according to the Declaration of Helsinki. All participants gave written informed consent before participation. The study was designed as a randomized, cross-over, controlled trial (Figure 1A). Participants were randomly assigned to group A or B. Group A was exposed to a cold room setting first, followed by a warm room setting, and group B vice versa. A climate chamber with controlled temperature settings was used as cold room setting and a medical examiner’s office as warm room setting.

**Figure 2:**
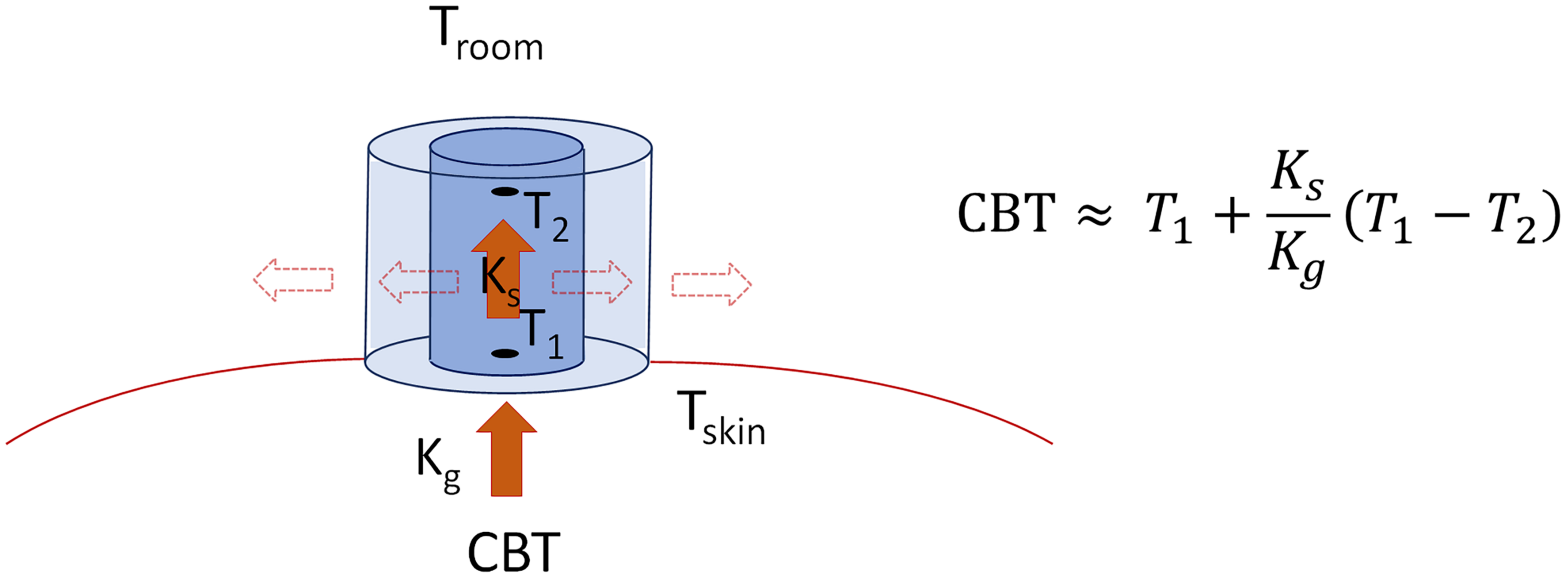
Schematic representation of the Double Sensor building blocks and core body temperature (CBT) estimation principles. The Double Sensor is composed by two temperature sensors (black dots), measuring temperatures *T*_1_ (approximating skin temperature *T*_skin_) and *T*_2_ (approximating ambient temperature *T*_amb_), respectively, which are separated by a disk of insulating material (in blue) of known heat transfer coefficient *K*_s_. Neglecting heat flux through lateral walls (dashed arrows), CBT can be estimated simply equating the heat flux from the body to the skin (with body heat transfer coefficient *K*_g_) to the heat flux from the skin through the sensor (red arrows), leading to the equation on the right.


As shown in Figure 1B, body temperature was measured in all participants at the forehead by the DS (T_DS_), in the esophagus (T_ES_), and in the ear canal (T_TYMP_). T_ES_ was used as an invasive reference measure of CBT, while T_TYMP_ was used as a non-invasive comparator. Probes were placed after at least 30 min of rest in the medical examiner’s office. CBT measurements with the DS were performed with a previously described sensor (Dräger, Lübeck, Germany). Details about the underlying biophysical model of the sensor are reported elsewhere (Gunga et al., 2008). Briefly, as shown in the schematic representation in Figure 2, the DS estimates CBT using two temperature sensors (black dots), positioned in proximity of the top and down surfaces of the device, and separated by an insulating disk (in blue). Assuming that: (1) the temperature T_1_ measured by the sensor adjacent to the bottom surface approximates skin temperature, (2) the temperature T_2_ measured by the sensor adjacent to the top surface approximates room temperature close to the surface, and (3) lateral heat loss from the device can be neglected, CBT can be estimated simply using the heat-flux equation and, specifically, by equating the heat flow from the body to the skin to that flowing through the insulating disk, which leads to:

(1) $$CBT\approx T_{1}+\frac{K_{s}}{K_{g}}\left(T_{1}-T_{2}\right)$$

where *K_s_* is the known heat transfer coefficient of the insulation disk containing the sensors, and *K_g_* is the heat transfer coefficient of the body. The presence of a heat flow through the DS lateral walls (dashed arrows in Figure 2), not included in Equation 1, may determine the underestimation of CBT, when heat leaves the DS, or to its overestimation when heat enters the DS (Gunga et al., 2008).

According to previous indications, the DS was positioned on the forehead at the vertical line above the eye directly underneath the hairline (Gunga et al., 2009). The sensor was applied using an elastic headband, ensuring continuous contact even in the presence of head movements. A small amount of conductive gel was applied between the sensor and the skin. T_DS_ was continuously monitored with the mobPhysioLab^®^ (KORA Industrie-Elektronik GmbH, Hambühren, Germany) and displayed simultaneously with T_ES_ and T_TYMP_. The tympanic probe (M1024233, GE Healthcare Finland Oy, Helsinki, Finland) was inserted into the right ear canal according to the producer’s instructions and fixed to the lobe using standard surgical tape to prevent displacement. The esophageal probe (9F general-purpose sterile probe M1024229, GE Healthcare Finland Oy, Helsinki, Finland) was inserted *via* the naris into the lower third of the esophagus after local anesthesia of the nasal and pharyngeal mucosa with topical lidocaine 2% solution. After successful insertion, the probes were connected to an intensive care monitor (Compact Anaesthesia Monitor, GE Healthcare Finland Oy, Helsinki, Finland).

After the placement of the probes, participants were guided to the first test setting. Participants were seated during the test duration and measurements were recorded every 2 s and averaged over 1 min for a total duration of 10 min in each condition. The interval to transfer to the second test setting and start data recording was between 3 min and 5 min. Winter clothing, including a hat (not covering the DS), was allowed during measurements at low ambient temperature.

### Statistical Analysis

Data were reported as mean ± standard deviation (SD). The Shapiro–Wilk test assessed data normality. To evaluate statistical differences between temperature measurements over time and in different ambient conditions, data were processed by ANOVA mixed models. Temperature differences over time were assessed for each body site and ambient condition (warm/cold) by fitting a mixed model (M1) with time (measurements at 3, 6, and 9 min) as within-subject factor and randomization group (A/B) as between-subject factor. Temperature differences among ambient conditions (warm vs. cold room) were assessed after a period of 9 min at each body site by fitting a mixed model with the ambient condition as within-subject factor and randomization group as a between-subject factor (model M2). In each model, the presence of factor interaction was expressed in terms of F-statistics and corrected values of *p* (Greenhouse–Geisser adjustment) to address sphericity deviations. Post-hoc tests were performed using Bonferroni correction for multiple comparisons.

DS capability in tracking CBT was assessed by comparing DS measurements with the esophageal and tympanic measurements. The bias between the T_DS_ and T_ES_/T_TYMP_ was evaluated by constructing Bland–Altman plots and by calculating the bias or mean difference (MD) and the limits of agreement (LoA, 1.96^*^SD) between DS and esophageal/tympanic measurements. The degree of the linear relationship between the T_DS_ and T_ES_/T_TYMP_ was quantified using Pearson’s correlation coefficient (r) and significance level. Lin’s concordance correlation coefficient (CCC) with confidence interval (CI) was also estimated as a cumulative measure of accuracy and precision. The analyses were performed for the warm and cold room settings, in the whole study group and in the two randomization subgroups (group A and B).

Statistical analyses were performed using STATISTICA (Version 7, Windows package) and Matlab (Version R2019b, MathWorks, Inc., Natick, MA, United States). A *p* < 0.05 was considered statistically significant.

## Results

### Study Group and Test Conditions

Twenty-one participants (mean age 39 ± 12 years, range 22–61 years, two females) were enrolled in the study. None of the participants dropped out. Ten subjects were assigned to group A and eleven subjects to group B. The temperature in the warm room setting was 23.2 ± 0.4°C and in the cold room setting was −18.7 ± 1.0°C.

### Time Course of Temperature Measurements Under Different Room Temperature

**Figure 3:**
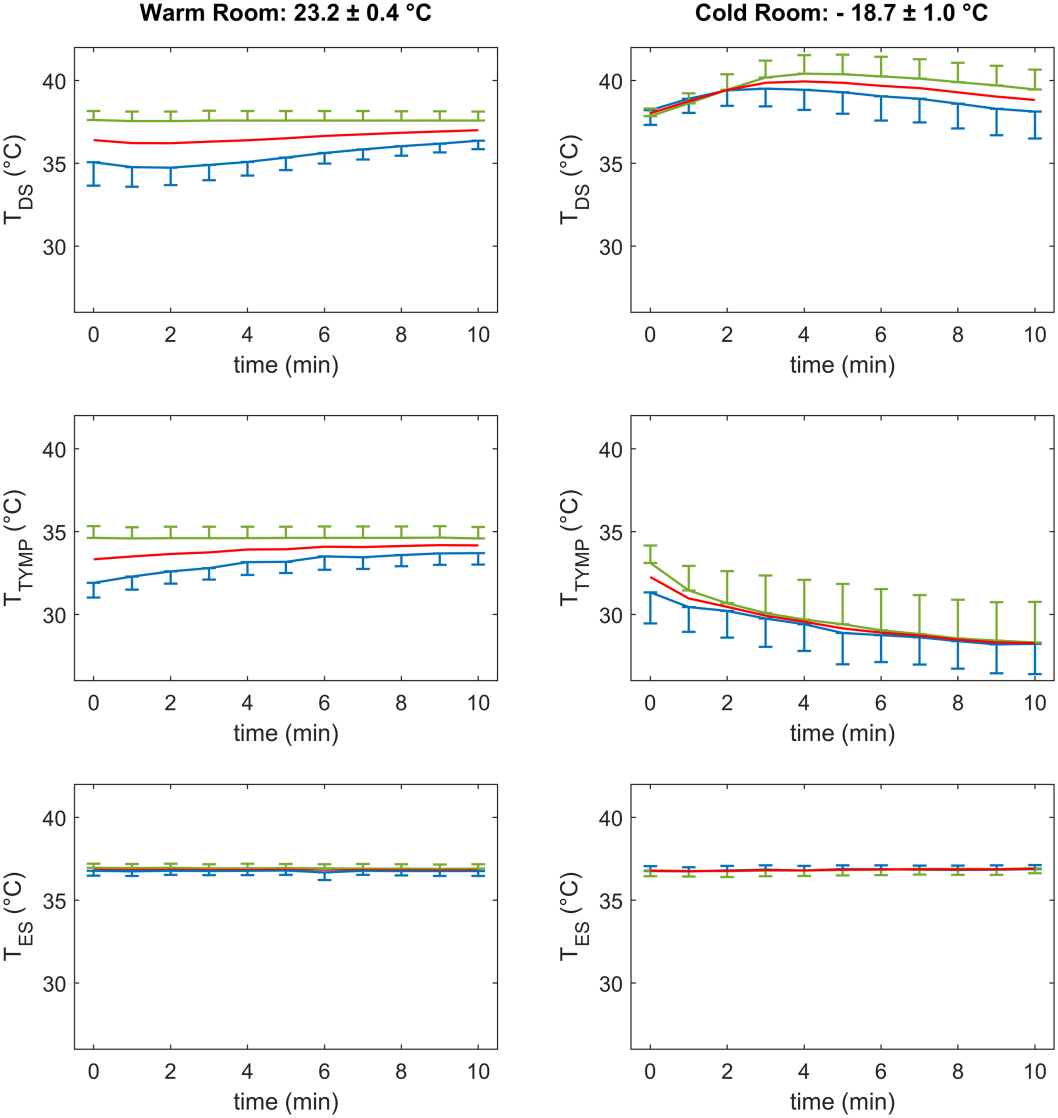
Time course of average temperature values measured by the forehead double sensor (T_DS_, upper panels), the tympanic sensor (T_TYMP_, central panels), and the esophageal sensor (T_ES_, bottom panels) in the warm (left) and cold (right) room settings in the overall population (red) and in group A (exposure to cold followed by warm room setting, blue) and group B (exposure to warm followed by cold room setting, green). Data are mean and standard deviation.

The time course of average temperature values recorded by the DS, tympanic, and esophageal sensors are displayed in Figure 3 for the warm (left panels) and cold room settings (right panels). Average temperature values measured by the three devices over time are summarized in Table 1.

**Table 1:** Time course of temperature values measured by the Double Sensor (T_DS_), tympanic (T_TYMP_), and esophageal sensors (T_ES_) in the warm and cold room settings for the whole population and in the two randomization groups (group A: exposure to cold followed by warm room setting; group B: exposure to warm followed by cold room setting).

**Warm room (*T* = 23.4 ± 0.4°C)**				
**Time**	**Groups**	**T**_**DS**_ **(°C)**	**T**_**TYMP**_ **(°C)**	**T**_**ES**_ **(°C)**
3 min	Overall	36.3 ± 1.6	33.8 ± 1.2	36.8 ± 0.2
	Group A	34.9 ± 0.9^*^	32.8 ± 0.7^*^	36.8 ± 0.2
	Group B	37.6 ± 0.6	34.6 ± 0.7	36.9 ± 0.2
6 min	Overall	36.7 ± 1.2^&^	34.1 ± 0.9^&^	36.8 ± 0.4
	Group A	35.6 ± 0.6^&^^,^^*^	33.5 ± 0.8^&^^,^^*^	36.7 ± 0.5
	Group B	37.6 ± 0.6	34.6 ± 0.7	36.9 ± 0.2
9 min	Overall	36.9 ± 0.9^&^^,^^£^	34.2 ± 0.8^&^	36.8 ± 0.3
	Group A	36.2 ± 0.5^&^^,^^£^^,^^*^	33.7 ± 0.7^&^^,^^*^	36.8 ± 0.3
	Group B	37.6 ± 0.5	34.6 ± 0.7	36.9 ± 0.2
M1 statistics		*F* = 74.4, *p* < 0.0001	*F* = 16.1, *p* = 0.0001	*F* = 0.95, *p* = 0.36
**Cold room (T = −18.7 ± 1.0°C)**				
**Time**	**Groups**	**T**_**DS**_ **(°C)**	**T**_**TYMP**_ **(°C)**	**T**_**ES**_ **(°C)**
3 min	Overall	39.9 ± 1.1	29.9 ± 2.0	36.8 ± 0.3
	Group A	39.5 ± 1.1	29.7 ± 1.7	36.8 ± 0.3
	Group B	40.2 ± 1.0	30.1 ± 2.3	36.8 ± 0.3
6 min	Overall	39.7 ± 1.4	28.9 ± 2.1^&^	36.9 ± 0.3
	Group A	39.0 ± 1.5^&^	28.7 ± 1.6	36.9 ± 0.2
	Group B	40.2 ± 1.2	29.0 ± 2.5	36.8 ± 0.3
9 min	Overall	39.0 ± 1.5^&^^,^^£^	28.3 ± 2.0^&^^,^^£^	36.9 ± 0.3
	Group A	38.3 ± 1.6^&^^,^^£^^,^^*^	28.2 ± 1.7	36.8 ± 0.3
	Group B	39.7 ± 1.2^£^	28.4 ± 2.3	36.9 ± 0.3
M1 statistics		*F* = 4.80, *p* = 0.03	*F* = 0.07, *p* = 0.88	*F* = 1.23, *p* = 0.30

Data are mean ± standard deviations.

& Post-hoc differences between time points: *p* < 0.05 versus 3 min.

£ Post-hoc differences between time points; *p* < 0.05 versus 6 min.

* Post-hoc differences between randomization groups; *p* < 0.05 versus group B.

DS-based temperature values of all participants (Figure 3, top left panel), measured in the warm room, changed from 36.3 ± 1.6°C at 3 min to 36.9 ± 0.9°C at 9 min, showing a progressive decrease in variability over time. As shown in Table 1, a significant interaction between time and randomization group was observed (*p* < 0.0001). Group A showed a more marked temporal trend, and higher variability than group B. T_DS_ in group A progressively and significantly increased from 3 min to 6 min to 9 min (*p* < 0.0001). In contrast, no significant differences between T_DS_ values at different time points were observed for group B (*p* = n.s.). Although differences between randomization groups tended to decrease over time, the two groups maintained a significant difference at all three time points (*p* < 0.0001), with group A showing significantly lower temperature values. In the cold environment (Figure 3, top right panel), the DS displayed a biphasic behavior, with an initial increase of temperature in the first three minutes followed by a decrease. Temperature measurement variability in the overall population increased over time from 1.1°C at 3 min to 1.5°C at 9 min. A significant interaction between time and randomization was observed also in this condition (*p* = 0.03). Group A showed significant differences from 3 min to 6 and 9 min and from 6 to 9 min (*p* < 0.0001), while group B showed a significant difference only between 6 and 9 min (*p* < 0.05). A progressive separation of the two randomization groups was observed, and a statistically significant difference was reached at 9 min (p < 0.05), where group A showed significantly lower values. At 9 min, a significant difference between T_DS_ values in the warm and cold room conditions was observed for the whole population (*p* < 0.0001).

T_TYMP_ values in the warm room setting (Figure 3, central left panel) displayed similar behavior to the DS, with comparable variability, time course, differences in the two randomization groups, and a significant interaction between the factors (*p* < 0.0001, Table 1). T_TYMP_ displayed a progressive decrease to low values in the cold room condition (Figure 3, central right panel), decreasing from 29.9 ± 2.0°C at 3 min to 28.3 ± 2.0°C at 9 min (*p* < 0.0001). The variability of T_TYMP_ remained high and almost constant over time. No marked difference was found between randomization groups (*p* = n.s.). At 9 min, a significant difference between the warm and cold room conditions was observed for the whole population (*p* < 0.0001).

Compared to the DS and tympanic sensors, CBT measurements by the esophageal probe (Figure 3, bottom panels) displayed very low variability at each time point (Table 1), stability over time, and no significant difference among randomization groups. Similar values were measured in the warm and cold room conditions in the overall population at 9 min (*p* = n.s.).

### Performance of the DS in Comparison With the Esophageal and Tympanic Sensors

**Figure 4:**
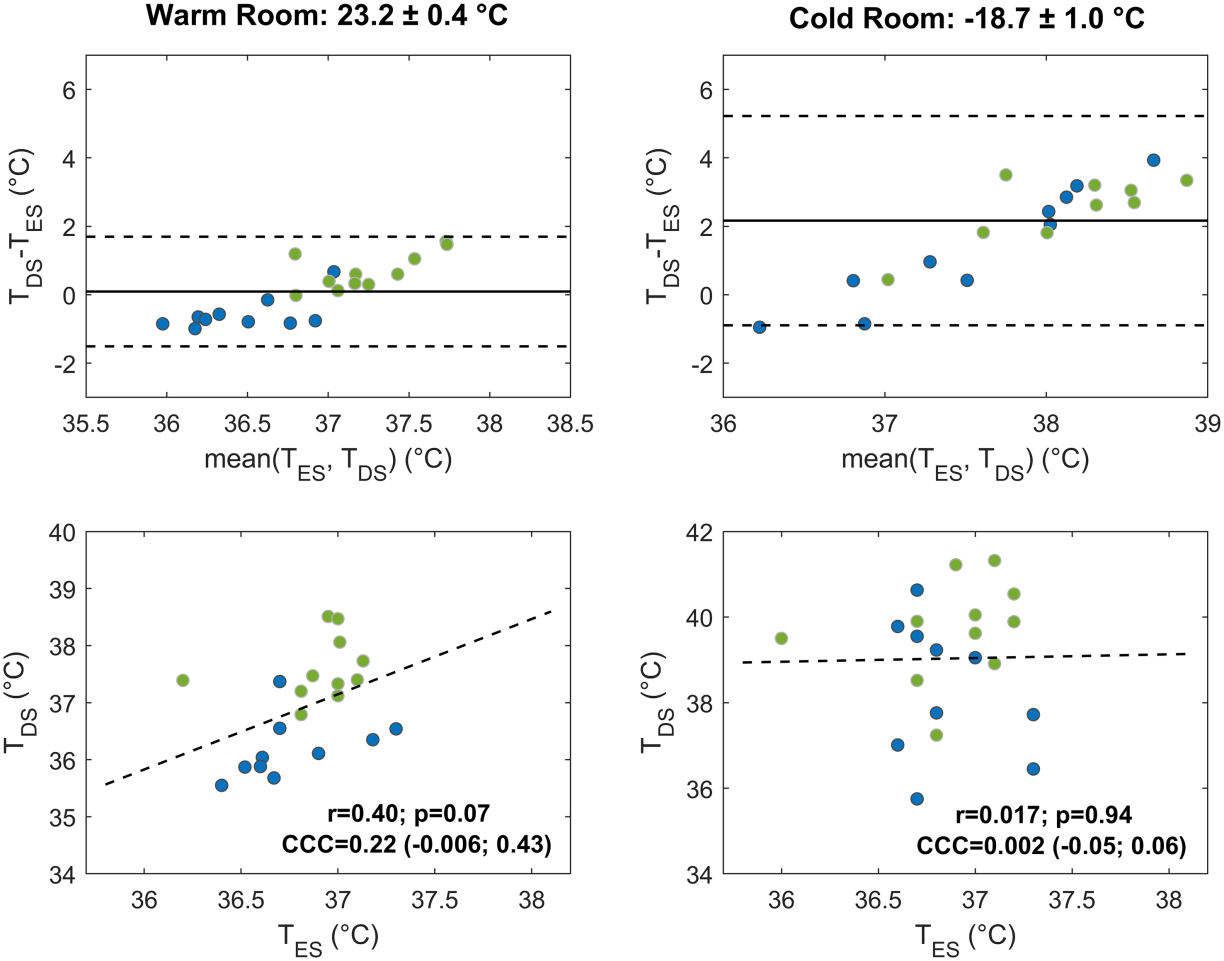
Agreement between temperature values measured by the forehead double sensor (T_DS_) and the esophageal sensor (T_ES_) after 9 min in the warm (left) and cold room (right) settings. Bland–Altman plots and correlation plots are displayed in the upper and lower panels, respectively, and subjects are color-coded according to the randomization groups (group A, blue; group B, green). In the Bland–Altman plots, the solid line indicates the mean difference and the dashed lines the limits of agreement (mean ± 1.96 standard deviation) for the overall population. In the correlation plots, the linear regression line (dashed line), Pearson’s correlation coefficient (r) with value of *p* (p), and Lin’s concordance correlation coefficient (CCC) and confidence interval, are indicated for the overall population.

**Figure 5:**
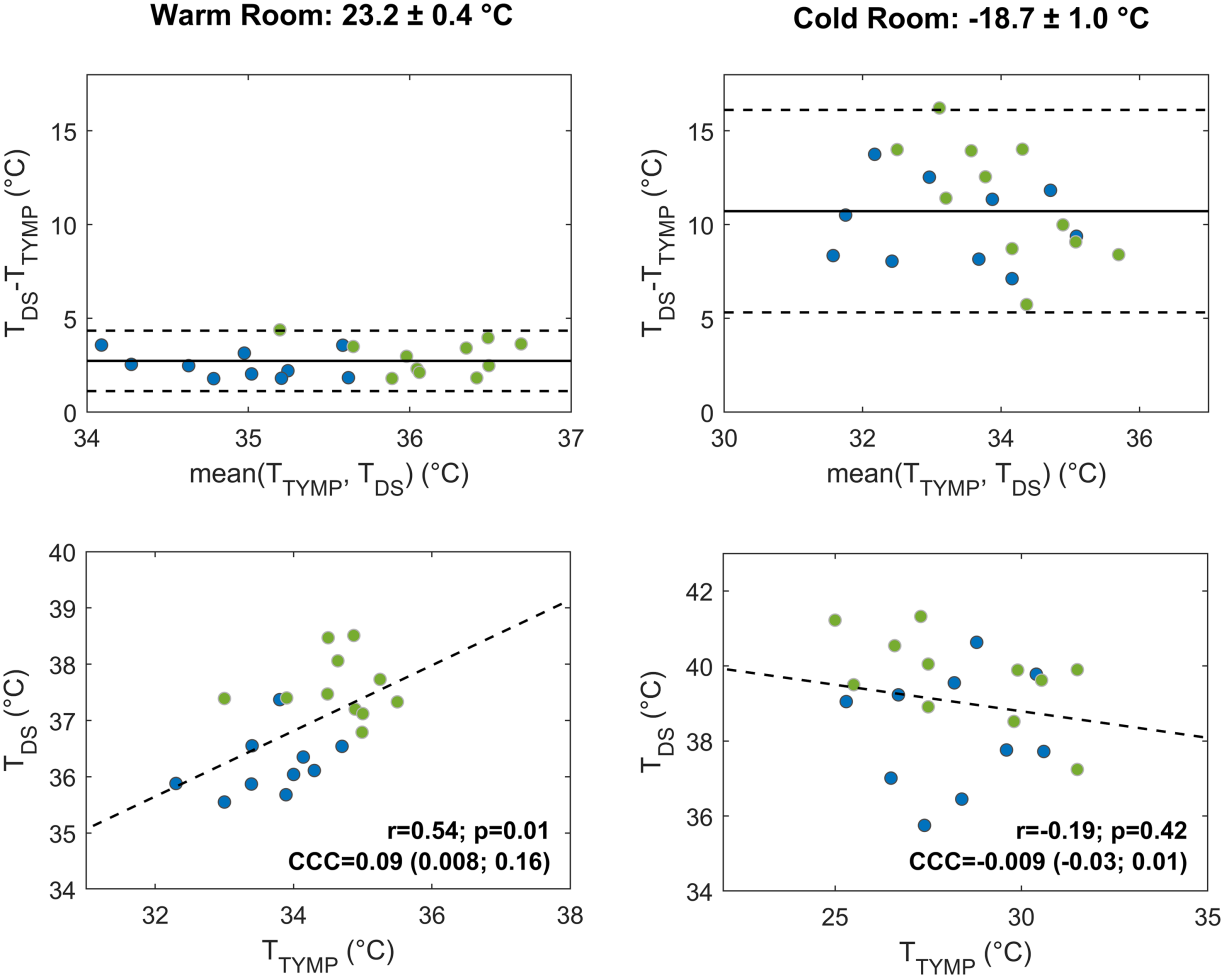
Agreement between temperature values measured by the forehead double sensor (T_DS_) and the tympanic sensor (T_TYMP_) after 9 min in the warm (left) and cold room (right) settings. Bland–Altman plots and correlation plots are displayed in the upper and in the lower panels, respectively, and subjects are color-coded according to the randomization groups (group A, blue; group B, green). In the Bland–Altman plots, the solid line indicates the mean difference and the dashed lines the limits of agreement (mean ± 1.96 standard deviation) for the overall population. In the correlation plots, the linear regression line (dashed line), Pearson’s correlation coefficient (r) with value of *p* (p), and Lin’s CCC and confidence interval, are indicated for the overall population.

The performance of the DS for estimating CBT under different environmental conditions and for the two randomization groups was evaluated in comparison with the esophageal and tympanic sensors. Results are summarized in Table 2 and Figures 4, 5.

**Table 2:** Agreement between the double sensor, esophageal, and tympanic sensors at different time points, room temperature settings, and study groups.

Warm room condition (T = 23.4 ± 0.4°C)							
**Time**	**Groups**	**Agreement with esophageal sensor**			**Agreement with tympanic sensor**		
		**r (p)**	**CCC (CI)**	**MD (LoA), °C**	**r (p)**	**CCC (CI)**	**MD (LoA), °C**
3 min	Overall	0.43 (<0.05)	0.12 (0.003; 0.24)	−0.54 (−3.42; 2.34)	0.76 (<0.0001)	0.26 (0.10; 0.40)	2.56 (0.58; 4.54)
	Group A	0.32 (0.37)	0.03 (−0.04; 0.10)	−1.85 (−3.55; −0.15)	0.34 (0.34)	0.07 (−0.08; 0.21)	2.11 (0.26; 3.96)
	Group B	0.21 (0.53)	0.07 (−0.15; 0.28)	0.65 (−0.48; 1.79)	−0.05 (0.89)	−0.004 (−0.05; 0.05)	2.97 (1.17; 4.78)
6 min	Overall	0.40 (0.07)	0.23 (−0.01; 0.45)	−0.15 (−2.25; 1.94)	0.62 (<0.005)	0.15 (0.03; 0.26)	2.56 (0.75; 4.38)
	Group A	0.24 (0.51)	0.08 (−0.15; 0.30)	−1.04 (−2.41; 0.33)	0.50 (0.14)	0.09 (−0.04; 0.21)	2.12 (0.68; 3.57)
	Group B	0.17 (0.61)	0.06 (−0.15; 0.26)	0.65 (−0.49; 1.79)	−0.06 (0.86)	−0.005 (−0.05; 0.04)	2.96 (1.15; 4.78)
9 min	Overall	0.40 (0.07)	0.22 (−0.006; 0.43)	0.09 (−1.51; 1.69)	0.54 (<0.05)	0.09 (0.008; 0.16)	2.74 (1.13; 4.35)
	Group A	0.42 (0.23)	0.18 (−0.12; 0.45)	−0.56 (−1.52; 0.39)	0.37 (0.30)	0.03 (−0.03; 0.10)	2.50 (1.12; 3.88)
	Group B	0.26 (0.45)	0.08 (−0.13; 0.28)	0.69 (−0.38; 1.76)	−0.03 (0.93)	−0.002 (−0.05; 0.05)	2.95 (1.19; 4.70)
Cold room condition (T = −18.7 ± 1.0°C)							
		**Agreement with esophageal sensor**			**Agreement with tympanic sensor**		
**Time**	**Groups**	**r (p)**	**CCC (CI)**	**MD (LoA), °C**	**r (p)**	**CCC (CI)**	**MD (LoA), °C**
3 min	Overall	0.10 (0.68)	0.006 (−0.02; 0.03)	3.05 (0.91; 5.18)	−0.24 (0.29)	−0.009 (−0.03; 0.009)	9.94 (5.10; 14.8)
	Group A	−0.28 (0.44)	−0.02 (−0.06; 0.03)	2.67 (0.36; 4.98)	0.21 (0.56)	0.007 (−0.02; 0.03)	9.76 (6.21; 13.3)
	Group B	0.43 (0.18)	0.02 (−0.01; 0.06)	3.39 (1.59; 5.19)	−0.64 (<0.05)	−0.02 (−0.06; 0.006)	10.1 (4.17; 16.0)
6 min	Overall	0.06 (0.79)	0.005 (−0.03; 0.04)	2.82 (0.01; 5.64)	−0.27 (0.24)	−0.01 (−0.03; 0.009)	10.8 (5.26; 16.3)
	Group A	−0.38 (0.27)	−0.03 (−0.10; 0.03)	2.18 (−0.89; 5.25)	0.09 (0.80)	0.004 (−0.02; 0.03)	10.3 (6.23; 14.4)
	Group B	0.47 (0.14)	0.03 (−0.01; 0.07)	3.41 (1.33; 5.48)	−0.67 (<0.05)	−0.03 (−0.06; 0.005)	11.2 (4.56; 17.9)
9 min	Overall	0.02 (0.94)	0.002 (−0.05; 0.06)	2.16 (−0.89; 5.22)	−0.19 (0.42)	−0.009 (−0.03; 0.01)	10.7 (5.32; 16.1)
	Group A	−0.32 (0.37)	−0.05 (−0.17; 0.07)	1.44 (−1.88; 4.77)	0.11 (0.76)	0.005 (−0.03; 0.04)	10.1 (5.75; 14.5)
	Group B	0.28 (0.41)	0.02 (−0.03; 0.07)	2.82 (0.60; 5.04)	−0.58 (0.06)	−0.02 (−0.05; 0.007)	11.3 (5.08; 17.5)

Data are given as Pearson’s correlation coefficient (r) with *p*-value (p), Lin’s concordance correlation coefficient (CCC) with confidence interval (CI), and mean difference or bias (MD) and limits of agreement (LoA, mean ± 1.96 standard deviation) between the forehead double sensor (DS) and the esophageal/tympanic sensors.

The DS displayed moderate agreement with esophageal and tympanic sensors in the warm room condition. The bias with the esophageal sensor was lower than that with the tympanic sensor, although confidence intervals were comparable in the two cases. Compared to the esophageal sensor, the bias showed a tendency to decrease over time, reaching a value of 0.09 (LoA: −1.5; 1.7) °C at 9 min. Group A slightly underestimated the temperature (bias of −0.6°C), and group B slightly overestimated it (0.7°C). Compared to the tympanic sensor, the DS showed a larger positive bias of 2.7 (LoA: 1.1; 4.3) °C at 9 min. Both randomization groups displayed higher T_DS_ values than T_TYMP_ with slightly worse performance and larger bias for group B (2.9°C). In the whole study group, the DS showed a moderate but not statistically significant correlation with the esophageal sensor (*r* = 0.4, *p* = 0.07 at 9 min) and a more robust, significant correlation with the tympanic sensor (*r* = 0.54, *p* < 0.05 at 9 min). When combining correlation and bias metrics at 9 min, the DS showed a fair agreement with the esophageal sensor [CCC = 0.22 (CI: −0.006; 0.43)] and a poor agreement with the tympanic sensor [CCC = 0.09 (CI: 0.008; 0.16)].

In the cold room condition, the agreement of the DS with both esophageal and tympanic sensors significantly decreased, especially with the latter sensor (Table 2). In terms of bias, a slight reduction of the bias with the esophageal sensor was observed over time, with relatively stable confidence intervals. At 9 min, the DS overestimated T_ES_ by 2.16°C with a large confidence interval (CI: −0.89; 5.22). Group A showed a smaller bias (1.4°C) than group B (2.8°C), but with slightly higher variability. The comparison with the tympanic sensor showed a larger bias and confidence interval. At 9 min, the bias reached a value of 10.7 (LoA: 5.3; 16.1) °C. Both groups showed higher T_DS_ values than T_TYMP_ values, with slightly smaller bias (10.1°C) and variability in group A than in group B (bias of 11.3°C). In the whole population, linear correlation with both sensors was lost. The increase of the bias and decrease of linear correlation led to a drop of Lin’s concordance correlation to almost-zero values.

## Discussion

This is the first study that analyzed the effects of low ambient temperature and ambient temperature changes on the accuracy of DS-based estimation of CBT in normothermic participants. The study was performed by exposing participants to warm and cold room conditions following a randomized cross-over design and comparing DS measurements with esophageal and tympanic measurements. Consistent with our initial hypothesis, our results demonstrated the significant deterioration of DS accuracy at low ambient temperature and the existence of differences between randomization groups, suggesting the effects of previous exposure to cold on subsequent DS measurements. Significant differences were found between temperature values measured by both DS and tympanic sensors at warm versus cold ambient temperature, while the esophageal temperature was almost unaffected by ambient temperature changes. This led to a significant increase in the bias and the loss of correlation between the DS and esophageal/tympanic probes. In the warm room setting, the bias of the DS with the reference esophageal sensor decreased over time to a value of 0.09°C at 9 min. In contrast, in the cold ambient setting, the bias increased to 2.16°C. In terms of randomization effects, DS measurements displayed a significant difference between randomization groups in the warm room condition, where groups A and B underestimated and overestimated esophageal reference, respectively. Overall, both groups showed a significant overestimation of the esophageal temperature in the cold room condition.

The observed differences may be due to technical factors related to DS technology and to the specific anatomical and physiological aspects of the monitoring site. From a technical point of view, fast changes in ambient temperature and exposition to cold temperature may produce alterations in the heat flow through the sensors and variations in lateral heat loss (Gunga et al., 2008). These may result in fast changes of the measured temperature, with a subsequent slow re-adaptation of the sensor to regain a steady-state condition. When room temperature changes suddenly, the DS temperature sensors are not in equilibrium. In particular, the temperature T_2_ of the sensor close to the top surface does not reflect ambient temperature. Since the top part of the DS is the most exposed to the external environment, T_2_ undergoes the fastest changes, and its dynamics initially drive the estimation of CBT by Equation 1. Consistently, the low values of T_DS_ observed in group A after passing from the cold to the warm room setting may be associated with a fast increase of T_2_ when exposed to the warm environment. The following slow increase toward more reliable values during the warm room condition, where the bias with the esophageal sensor decreased from −1.85°C to −0.56°C, may be the result of the slow adaptation of the rest of the system to the new conditions. In a specular way, the fast increase in T_DS_ in both groups when passing from the warm (or pre-test) condition to the cold setting may be driven by the rapid drop in T_2_ in the cold environment. The delayed tendency to decrease during cold exposure may again result from a slow adaptation of the system. In addition to heat dynamics at the top DS surface, heat loss from the lateral wall is expected to be increased in the cold setting due to the increasing temperature gradient with the environment. Increased heat loss may have contributed, as a secondary factor, to the observed delayed decrease of T_DS_. In the warm setting, the progressive warming of the lateral surface of the DS may have led to reduced heat loss and thus to an increase of the measured T_DS_ (i.e., reduced underestimation). To mitigate the impact of sudden environmental temperature changes on DS temperature monitoring, additional insulating layers may be inserted between the upper temperature sensor and the top surface of the DS or a buffering zone of warm air may be created around the DS by inserting the device within a helmet (Gunga et al., 2008). This would help avoiding abrupt temperature transitions in the exposed DS regions and T_2_ overshoot, and it would promote a global adaptation of the device to the new condition. The worse agreement between T_DS_ and T_ES_ and the augmented T_DS_ in the cold room setting are consistent with previous studies. A reduction in the accuracy of DS measurements compared to rectal CBT was observed in a set of experiments, where the ambient temperature was reduced from 25°C/40°C to 10°C (Gunga et al., 2008). A progressive increase in DS temperature was observed in parachutists during free fall and interpreted in the light of meteorological conditions (i.e., effects of wind blow on the sensor; Werner et al., 2013). Although in the opposite direction, the tympanic sensor also displayed a worsened performance in the cold condition. T_TYMP_ experienced a substantial decrease over time when exposed to the cold room, resulting in a loss of correlation and an increased bias (10.7°C at 9 min) between the tympanic sensor and the DS. This is consistent with previous works showing the detrimental effects of cold environments on tympanic measurements (Gagnon et al., 2010; Teunissen et al., 2011; Skaiaa et al., 2015; Strapazzon et al., 2015). Of note, these works demonstrated that the effects of cold could be reduced when insulation was applied to protect the sensor, as we suggested here to improve DS performance.

In addition to technical factors, differences or similarities in temperature dynamics among body sites in diverse conditions may be partially explained in the light of physiological factors. A higher cooling rate characterizes the forehead site—combined with a very high perfusion rate—compared to invasive locations, with the head acting as a thermal window through its extraordinary network of veins (scalp, emissary, and diploic veins; Nunneley et al., 1982; Hershkovitz et al., 1999). In the cold environment, peripheral vasoconstriction can slowly build up, increasing body insulation and decreasing skin temperature to preserve core temperature (Castellani and Young, 2016). The rise of vasoconstriction and increased insulation can be roughly modeled in terms of a decrease in the heat transfer coefficient of the body. Since body thermal conductivity, *K_g_*, is assumed constant in Equation 1, T_DS_ may slightly underestimate CBT values when vasoconstriction occurs. Thus, this factor may have partially contributed to the delayed decreasing trend of T_DS_ observed in the cold environment. The asymmetric behavior at different ambient temperatures associated with the randomization procedure may also be partially attributed to physiological aspects. The effect of the warm/cold condition order on CBT may be related to the amount of work done in absorbing and dissipating heat (Parkhurst, 2010; Opatz et al., 2013). It has been shown that the gradient among different sensor positions is influenced by vasomotor responses (Parkhurst, 2010), which are not simply determined by instantaneous thermal input to central controllers, but may also depend on the direction of core temperature changes and skin temperature perception (Ozaki et al., 1993; Sessler, 1993; Kiyatkin, 2018). Following these assumptions and previous studies, it is possible that the low local cutaneous temperature experienced in group A prolonged—in the warm room condition—the shunt constriction induced by a centrally mediated drive to reduce heat dissipation (Ozaki et al., 1993). On the other hand, the significant correlation observed between the DS and the tympanic probe in the warm room may be attributed to the fact that the vasculature patterns supplying both the ear canal and head skin derive from the external and internal carotid arteries (Benzinger and Taylor, 1963; McCarthy and Heusch, 2006), which may favor thermal equilibrium between the two sites.

## Limitations

The study was performed on healthy and normothermic subjects. Results cannot be extrapolated to patients with acute and/or chronic pathological states (for instance, affecting blood flow and peripheral circulation). The lack of coverage of the DS may have contributed to the worsening of its performance in the cold room setting. Protection of the sensor with proper insulation [e.g., positioning inside a helmet as in (Gunga et al., 2008)] may help limiting environmental effects and improving performance. Correction terms to the model in Equation1 may also be considered to reduce bias due to increased heat loss (Gunga et al., 2008) in the cold or due to physiological changes and vasoconstriction. The study protocol was composed of two 10 min exposition windows separated by a short transfer time of 3–5 min. Given the observed long re-equilibration time of the DS, further studies with extended exposition and recovery periods should be performed for a thorough assessment of the DS response and performance compared with other sites for core temperature monitoring.

## Conclusion

We showed a significant deterioration of DS performance in estimating CBT at very low ambient temperatures. DS-based temperature values showed lower bias with the esophageal temperature at room ambient temperature, which may open the possibility of continuous recording under stable conditions. Nevertheless, the effects of the previous exposure to cold settings require that an adequate re-equilibration period is granted to obtain reliable measurements, precluding applications in the presence of fast temperature dynamics and environmental temperature changes. Integrating the DS sensor into helmets or covering it with a cap might help reducing the effects of cold air on the sensor and lateral heat loss, potentially improving performance. However, future studies and parallel technological developments are needed to allow in-field temperature monitoring by the DS under cold and/or changing environmental settings.

## Data Availability Statement

The datasets generated and analyzed in this study are available from the corresponding author on reasonable request.

## Ethics Statement

The study was reviewed and approved by Ethics Committee review board of Bolzano, Italy. The patients/participants provided their written informed consent to participate in this study.

## Author Contributions

AW, HB, GA, and GS conceived and designed the study. GP, GA, HB, and GS collected the data. MM, AM, GS, and MF performed data analysis. AM, MM, and GS wrote the manuscript. All authors contributed to the manuscript critical revision, read, and approved the submitted version.

## Conflict of Interest

The authors declare that the research was conducted without any commercial or financial relationships that could be construed as a potential conflict of interest.

## Publisher’s Note

All claims expressed in this article are solely those of the authors and do not necessarily represent those of their affiliated organizations, or those of the publisher, the editors and the reviewers. Any product that may be evaluated in this article, or claim that may be made by its manufacturer, is not guaranteed or endorsed by the publisher.

## References

[ref1] Asadian S. Khatony A. Moradi G. Abdi A. Rezaei M. (2016). Accuracy and precision of four common peripheral temperature measurement methods in intensive care patients. Med Dev. 9, 301–308. doi: 10.2147/MDER.S109904, PMID: 27621673 PMC5012839

[ref2] Aschoff J. Wever R. (1958). Kern und Schale im Wärmehaushalt des Menschen. Naturwissenschaften 45, 477–485. doi: 10.1007/BF00635546

[ref3] Benzinger T. H. Taylor G. W. (1963). “Cranial measurements of internal temperature in man,” in Temperature – Its Measurement and Control in Science and Industry. ed. C. M. Herzfeld (New York: Reinhold), 111–120.

[ref4] Brinnel H. Cabanac M. (1989). Tympanic temperature is a core temperature in humans. J. Therm. Biol. 14, 47–53. doi: 10.1016/0306-4565(89)90029-6

[ref5] Castellani J. W. Young A. J. (2016). Human physiological responses to cold exposure: acute responses and acclimatization to prolonged exposure. Auton. Neurosci. 196, 63–74. doi: 10.1016/j.autneu.2016.02.009, PMID: 26924539

[ref6] Fox R. H. Solman A. J. (1971). A new technique for monitoring the deep body temperature in man from the intact skin surface. J. Physiol. 212, 8–10. 5548025

[ref7] Fulbrook P. (1997). Core body temperature measurement: a comparison of axilla, tympanic membrane and pulmonary artery blood temperature. Intensive Crit. Care Nurs. 13, 266–272. doi: 10.1016/s0964-3397(97)80425-9, PMID: 9538713

[ref8] Gagnon D. Lemire B. B. Jay O. Kenny G. P. (2010). Aural canal, esophageal, and rectal temperatures during exertional heat stress and the subsequent recovery period. J. Athl. Train. 45, 157–163. doi: 10.4085/1062-6050-45.2.157, PMID: 20210619 PMC2838467

[ref9] Gallimore D. (2004). Reviewing the effectiveness of tympanic thermometers. Nurs. Times 100, 32–34. PMID: . 15373157

[ref10] Gómez-Romero F. J. Fernández-Prada M. Fernández-Suárez F. E. Gutiérrez-González C. Estrada-Martínez M. Cachero-Martínez D.. (2019). Intra-operative temperature monitoring with two non-invasive devices (3M Spoton^®^ and Dräger Tcore^®^) in comparison with the swan-Ganz catheter. Cirugía Cardio. 26, 191–196. doi: 10.1016/j.circv.2019.06.002

[ref11] Gunga H.-C. Sandsund M. Reinertsen R. E. Sattler F. Koch J. (2008). A non-invasive device to continuously determine heat strain in humans. J. Therm. Biol. 33, 297–307. doi: 10.1016/j.jtherbio.2008.03.004

[ref12] Gunga H.-C. Werner A. Stahn A. Steinach M. Schlabs T. Koralewski E.. (2009). The double sensor-A non-invasive device to continuously monitor core temperature in humans on earth and in space. Respir. Physiol. Neurobiol. 169, S63–S68. doi: 10.1016/j.resp.2009.04.005, PMID: 19428314

[ref13] Guschlbauer M. Maul A. C. Yan X. Herff H. Annecke T. Sterner-Kock A.. (2016). Zero-heat-flux thermometry for non-invasive measurement of Core body temperature in pigs. PLoS One 11:e0150759. doi: 10.1371/journal.pone.0150759, PMID: 26938613 PMC4777531

[ref14] Hershkovitz I. Greenwald C. Rothschild B. M. Latimer B. Dutour O. Jellema L. M.. (1999). The elusive diploic veins: anthropological and anatomical perspective. Am. J. Phys. Anthropol. 108, 345–358. doi: 10.1002/(SICI)1096-8644(199903)108:3<345::AID-AJPA9>3.0.CO;2-S, PMID: 10096685

[ref15] Janke D. Kagelmann N. Storm C. Maggioni M. A. Kienast C. Gunga H.-C.. (2021). Measuring Core body temperature using a non-invasive, disposable double-sensor During targeted temperature Management in Post-cardiac Arrest Patients. Front. Med. 8:666908. doi: 10.3389/fmed.2021.666908, PMID: 34026794 PMC8132874

[ref16] Kimberger O. Saager L. Egan C. Sanchez I. P. Dizili S. Koch J.. (2013). The accuracy of a disposable noninvasive core thermometer. Can. J. Anaesth. 60, 1190–1196. doi: 10.1007/s12630-013-0047-z, PMID: 24214518

[ref17] Kimberger O. Thell R. Schuh M. Koch J. Sessler D. I. Kurz A. (2009). Accuracy and precision of a novel non-invasive core thermometer. Br. J. Anaesth. 103, 226–231. doi: 10.1093/bja/aep134, PMID: 19482858

[ref18] Kiyatkin E. A. (2018). Brain temperature: from physiology and pharmacology to neuropathology. Handb. Clin. Neurol. 157, 483–504. doi: 10.1016/B978-0-444-64074-1.00030-6, PMID: 30459022

[ref19] Kobayashi T. Nemoto T. Kamiya A. Togawa T. (1975). Improvement of deep body thermometer for man. Ann. Biomed. Eng. 3, 181–188. doi: 10.1007/BF02363069, PMID: 1211681

[ref20] Lankford H. V. Fox L. R. (2021). The wind-chill index. Wilderness Environ. Med. 32, 392–399. doi: 10.1016/j.wem.2021.04.005, PMID: 34294536

[ref21] Lim C. L. Byrne C. Lee J. K. (2008). Human thermoregulation and measurement of body temperature in exercise and clinical settings. Ann. Acad. Med. 37, 347–353. 18461221

[ref22] Masè M. Micarelli A. Falla M. Regli I. B. Strapazzon G. (2021). Insight into the use of tympanic temperature during target temperature management in emergency and critical care: a scoping review. J. Intensive Care 9:43. doi: 10.1186/s40560-021-00558-4, PMID: 34118993 PMC8199814

[ref23] Masè M. Micarelli A. Strapazzon G. (2020). Hearables: new perspectives and pitfalls of in-ear devices for physiological monitoring. A scoping Review. Front. Physiol. 11:568886. doi: 10.3389/fphys.2020.568886, PMID: 33178038 PMC7596679

[ref24] Mazgaoker S. Ketko I. Yanovich R. Heled Y. Epstein Y. (2017). Measuring core body temperature with a non-invasive sensor. J. Therm. Biol. 66, 17–20. doi: 10.1016/j.jtherbio.2017.03.007, PMID: 28477905

[ref25] McCarthy P. W. Heusch A. I. (2006). The vagaries of ear temperature assessment. J. Med. Eng. Technol. 30, 242–251. doi: 10.1080/03091900600711415, PMID: 16864236

[ref26] Mendt S. Brauns K. Friedl-Werner A. Belavy D. L. Steinach M. Schlabs T.. (2021). Long-term bed rest delays the circadian phase of Core body temperature. Front. Physiol. 12:658707. doi: 10.3389/fphys.2021.658707, PMID: 34040542 PMC8141791

[ref27] Mendt S. Maggioni M. A. Nordine M. Steinach M. Opatz O. Belavý D.. (2017). Circadian rhythms in bed rest: monitoring core body temperature via heat-flux approach is superior to skin surface temperature. Chronobiol. Int. 34, 666–676. doi: 10.1080/07420528.2016.1224241, PMID: 27726448

[ref28] Moran D. S. Mendal L. (2002). Core temperature measurement: methods and current insights. Sports Med. 32, 879–885. doi: 10.2165/00007256-200232140-00001 12427049

[ref29] Muravchick S. (1983). Deep body thermometry during general anesthesia. Anesthesiology 58, 271–274. doi: 10.1097/00000542-198303000-00014, PMID: 6829963

[ref30] Nunneley S. A. Reader D. C. Maldonado R. J. (1982). Head-temperature effects on physiology, comfort, and performance during hyperthermia. Aviat. Space Environ. Med. 53, 623–628. 7115249

[ref31] Opatz O. Trippel T. Lochner A. Werner A. Stahn A. Steinach M.. (2013). Temporal and spatial dispersion of human body temperature during deep hypothermia. Br. J. Anaesth. 111, 768–775. doi: 10.1093/bja/aet217, PMID: 23801744

[ref32] Ozaki M. Sessler D. I. McGuire J. Blanchard D. Schroeder M. Moayeri A. (1993). The direction dependence of thermoregulatory vasoconstriction during isoflurane/epidural anesthesia in humans. Anesth. Analg. 77, 811–816. doi: 10.1213/00000539-199310000-00027, PMID: 8214670

[ref33] Parkhurst A. M. (2010). Model for understanding thermal hysteresis during heat stress: a matter of direction. Int. J. Biometeorol. 54, 637–645. doi: 10.1007/s00484-009-0299-z, PMID: 20140629

[ref34] Sakuragi T. Mukai M. Dan K. (1993). Deep body temperature during the warming phase of cardiopulmonary bypass. Br. J. Anaesth. 71, 583–585. doi: 10.1093/bja/71.4.583, PMID: 8260310

[ref35] Sessler D. I. (1993). Perianesthetic thermoregulation and heat balance in humans. FASEB J. 7, 638–644. doi: 10.1096/fasebj.7.8.8500688, PMID: 8500688

[ref36] Skaiaa S. C. Brattebø G. Aßmus J. Thomassen Ø. (2015). The impact of environmental factors in pre-hospital thermistor-based tympanic temperature measurement: a pilot field study. Scand. J. Trauma Resusc. Emerg. Med. 23:72. doi: 10.1186/s13049-015-0148-5, PMID: 26400226 PMC4581419

[ref37] Stahn A. C. Werner A. Opatz O. Maggioni M. A. Steinach M. von Ahlefeld V. W.. (2017). Increased core body temperature in astronauts during long-duration space missions. Sci. Rep. 7:16180. doi: 10.1038/s41598-017-15560-w, PMID: 29170507 PMC5701078

[ref38] Strapazzon G. Procter E. Paal P. Brugger H. (2014). Pre-hospital core temperature measurement in accidental and therapeutic hypothermia. High Alt. Med. Biol. 15, 104–111. doi: 10.1089/ham.2014.1008, PMID: 24950388

[ref39] Strapazzon G. Procter E. Putzer G. Avancini G. Dal Cappello T. Überbacher N.. (2015). Influence of low ambient temperature on epitympanic temperature measurement: a prospective randomized clinical study. Scand. J. Trauma Resusc. Emerg. Med. 23:90. doi: 10.1186/s13049-015-0172-5, PMID: 26542476 PMC4635596

[ref40] Taylor N. A. S. Tipton M. J. Kenny G. P. (2014). Considerations for the measurement of core, skin and mean body temperatures. J. Therm. Biol. 46, 72–101. doi: 10.1016/j.jtherbio.2014.10.006, PMID: 25455943

[ref41] Teunissen L. P. J. Klewer J. De Haan A. De Koning J. J. Daanen H. A. M. (2011). Non-invasive continuous core temperature measurement by zero heat flux. Physiol. Meas. 32, 559–570. doi: 10.1088/0967-3334/32/5/005, PMID: 21444968

[ref42] Werner A. Naschold U. Wilke T. Gunga H. (2013). Sauerstoffversorgung in operationellen Höhlen bei Fallschirmspringern – Studie zum Monitoring Psyhiologischer – Parameter in der realem Umwelt. Available at: https://wehrmed.de/humanmedizin/sauerstoffversorgung-in-operationellen-hoehlen-bei-fallschirmspringern-studie-zum-monitoring-psyhiologischer-parameter-in-der-realen-umwelt.html (Accessed September 29, 2021).

[ref43] Xu X. Karis A. J. Buller M. J. Santee W. R. (2013). Relationship between core temperature, skin temperature, and heat flux during exercise in heat. Eur. J. Appl. Physiol. 113, 2381–2389. doi: 10.1007/s00421-013-2674-z, PMID: 23775374

[ref44] Zeiner A. Klewer J. Sterz F. Haugk M. Krizanac D. Testori C.. (2010). Non-invasive continuous cerebral temperature monitoring in patients treated with mild therapeutic hypothermia: an observational pilot study. Resuscitation 81, 861–866. doi: 10.1016/j.resuscitation.2010.03.018, PMID: 20398992

